# Chemical Reactions, Drowning Swimmers, Owner's Manuals: The Power of Metaphors in Couple Therapy

**DOI:** 10.1111/famp.70120

**Published:** 2026-02-17

**Authors:** Arthur C. Nielsen

**Affiliations:** ^1^ Psychiatry and Behavioral Sciences Northwestern University Evanston Illinois USA; ^2^ The Chicago Psychoanalytic Institute and The Institute for Clinical Social Work Chicago Illinois USA

**Keywords:** couple therapy, mechanisms of change, metaphors, psychodynamics, therapeutic techniques

## Abstract

Over many years of working with couples, I have found that certain metaphors—drawn from images, films, stories, jokes, song lyrics, research findings, or events in my life—can be especially effective and memorable in clarifying and normalizing the diverse experiences of distressed couples. By likening events in therapy to more familiar situations, I have been able to strengthen the therapeutic alliance in a setting that otherwise might seem foreign, distressing, and even threatening. More broadly, metaphors have enriched my therapeutic repertoire, enabling me to help couples experience each other as more loving and mutually supportive. While many others have made this discovery and offered telling metaphors applicable to specific client problems (depression, procrastination, sexual dysfunction), this paper is unique as it provides metaphors targeted to help clients and students better comprehend the complex theories (systemic, psychodynamic, behavioral‐psychoeducational) that underlie our work. Following a brief literature review, I describe metaphors that have proved especially useful to accomplish that goal. Having set the stage with these examples, I then explore the topic more broadly, outline the multiple benefits of employing metaphors in therapy and offer technical suggestions for their effective use.

We tend to see metaphor as literary embroidery, what the poet Mark Doty calls “frosting on the cake of sense.” But really it is just language in its natural state, *especially when it is trying to gauge the unknown by setting it against the known*. [italics added].

Joe Moran (2019, 53).

*This brings us to one of the key functions of stories: to connect people and to transmit history and necessary lessons or skills for survival and adaptation. Human beings are storytelling animals*.

Nancy Kulish (2022, 831).

*For the most part, our comprehension of love is metaphorical, and we understand it primarily in terms of concepts for other natural kinds of experience: JOURNEYS, MADNESS, WAR, HEALTH, etc*.

Lakoff and Johnson (1980, 119).

## 1 Introduction

Chemical reactions, drowning swimmers, and owner's manuals, what do these have to do with couple therapy interventions? Everything! Over many years of working with couples (Nielsen 2016, 2017a, 2017b, 2022, 2023, 2024), I have found that certain metaphors like those just listed—ones drawn from images, films, stories, jokes, song lyrics, research findings, and events in my life—have been especially effective and memorable in clarifying and normalizing the diverse experiences of distressed couples. And because humans are wired to be receptive to stories (Gottschall 2012), narratives and metaphors have special power when we attempt to help couples improve their relationships and comprehend the process of therapy.

After briefly summarizing the relevant literature, I will describe metaphors that have proved especially useful to me and others both in clinical work and when teaching students. While many others have contributed to this subject and provided telling examples applicable to specific client problems (depression, procrastination, sexual dysfunction), this paper is unique as it provides metaphors targeted to help clients and students better comprehend the complex theories (systemic, psychodynamic, behavioral‐psychoeducational) that underlie our work.

After setting the stage with specific examples, I will summarize the intertwined benefits of using metaphors in therapy and make some technical recommendations on how best to use them. Overall, I will make the case that metaphors are essential to clinical competencies on the foundation of vast preexisting work and my 50 years of clinical experience.

## 2 Background, Literature, and Definitions

Since the seminal work of the linguists Lakoff and Johnson (1980), metaphors are no longer viewed as mere rhetorical flourishes, but as central to human thought, language, and emotional experience. As such, the modern understanding of metaphor is complex and lies at the intersection of linguistics, philosophy, anthropology, poetry, rhetoric, dream interpretation, hermeneutics, social constructivism, neurobiology, cognitive science, psychology broadly, and—relevant here—psychotherapy.

The literature on metaphors in *psycho*therapy is vast and full of interesting case examples. I have benefitted from reading much of it. Torneke (2017) in his review concluded that “Regardless of the specific treatment model, everyone seems to agree on the importance of metaphor as a therapeutic tool” (p. 2). Toward the end of this paper, I will have more to say about these areas of agreement. As concerns more formal research, a recent review of psychotherapy research on the topic (McMullen and Tay 2023) found many studies where clients reported greater benefits when therapists used metaphors, in keeping with a study showing that oncologists who used more metaphors received higher patient ratings (Casarett et al. 2010). Of course, it matters whether the metaphor fits the clinical situation, but both the clinical and more limited formal research literature point to the power of metaphors in clinical practice.

Readers looking for more on this topic will enjoy the following sources, which, taken together, show how the study of metaphor has become a pervasive and rewarding intellectual activity among psychotherapists of many orientations (Aragno 2009; Atwood and Levine 1991; Barker 1985; Burns 2001, 2007; Gottschall 2012; Katz 2013; Lankton and Lankton 1989; Papp 2014; Rosenblatt 1994; Torneke 2017).

The term *metaphor* comes from the ancient Greek *metaphorá*, meaning “transfer” or “carrying over,” so that a metaphor is a figure of speech in which a word or phrase from one domain (the source) is used to describe something in a different domain (the target) by highlighting shared characteristics. For example, Zhou et al. (2017) write:

In the conceptual metaphor “marriage is a journey,” the target domain is “marriage,” and the source domain is “journey.” The metaphor implies the following mappings: The couple correspond to travelers. The couples' relationship is understood as companionship in traveling. The couple has one destination and as travelers they share the same goal. The difficulties in the relationship correspond to the impediments to travel. (p. 67)

The value of metaphors lies in their ability to make sense of complex or unfamiliar experiences by describing them in terms of something else that is familiar. Metaphors put experience “into new words,” enabling us to conceptualize familiar things in unfamiliar ways and unfamiliar things in familiar ways. They often challenge entrenched or limiting conceptions by reframing them.

In this paper, following the cited authors and therapeutic traditions, I use *metaphor* broadly to encompass all instances where one thing is used to represent another. This inclusive definition, sometimes referred to as “figurative,” allows us to see metaphors in parables, fairy tales, films, therapist‐generated stories (Barker 1985; Burns 2001, 2007), narrative couple and family therapy (Combs and Freedman 1990; Skerrett 2022; White 2007), family sculpting (Papp et al. 2013), play therapy (Haen 2020; Lankton and Lankton 1989), art therapy (“a picture is worth a thousand words”), personal anecdotes, and more—all aimed at helping clients view their experience from new perspectives.

Like the authors cited above, I have spent years collecting and testing helpful metaphors. Discovering that others had done the same provided a kind of interrater reliability concerning the value of doing so and inspired me to share selections from my own extensive collection.

As I will discuss more later, some metaphors originate from the therapist, others from clients, and some are co‐created. Ones coming from therapists can be termed “prescriptive”—some of which are “stock” and ready to go, while others are created in the moment. These, in turn, can either target a particular patient problem (say sexual desire by comparing what inhibits or facilitates it to automobile brakes or accelerators) or a therapeutic concept (like transference by comparing it to a hidden map).^1^ While much of the literature focuses on the first category—for instance, Burns (2001, 2007), where you can find a story or parable for almost any problem—this paper centers, for the most part, on the second subcategory—*metaphors a couple therapist can have* “ready to hand” *to facilitate understanding of concepts central to cure*. We next examine such metaphors, grouped as they shed light on the three overarching approaches to couple therapy: systemic, psychodynamic, and behavioral and psychoeducational (Nielsen 2016, 2017a). This is the heart of my paper and most readers should find it *easier sledding*, practically valuable, and, at times, even fun to read.

## 3 Systemic Metaphors

Maladaptive couple processes are a hallmark of distressed relationships and one of the best predictors of marital decline, making them a central target for couple therapy (Gottman et al. 1998; Lebow et al. 2012). As we begin this discussion, note how the *technical language* of our field often borrows from cybernetics, architecture, and governance to characterize partner interactions. Terms like *structure, process, feedback, homeostasis, hierarchy, polarization*, and *cycles* (vicious or vulnerable) all suggest that patterned, repetitive interactions are more than just the sum of the individual psychologies of the partners. This *field theory* helps reframe and externalize couple problems, reducing defensiveness and blame while emphasizing that marital issues are typically, if not always, co‐created. Metaphors in this section aim to illuminate and improve couple processes.

### 3.1 *Lessons*: Setting the Stage^2^

Early in therapy, I often compare conjoint couple sessions to taking *lessons in music, sports, or dance*. I say, “If you are learning piano, tennis, or ballroom dancing, it's not sufficient to tell your teacher or coach what you do. He or she must watch you do it to help you improve. Similarly, having you talk to each other while I observe and try to help is the starting point for repairing and strengthening your relationship.”

This lessons metaphor allows me to frame my role as that of instructor, teacher, or coach—someone who creates a safe space where clients can take risks on their way to becoming proficient and, eventually, *playing on their own*. Sometimes I use the related metaphor of a *personal trainer*, someone who meets with you regularly and nudges you beyond your comfort zone. When using these images, I draw on my own experiences with coaches and therapists—times when I had to expose ingrained problems, and trust that I would be safe. The *lessons* metaphor conveys a hopeful sense of guidance and support from me as an experienced teacher. It contrasts sharply with one that clients often harbor semiconsciously: me as *a judge in a marital courtroom*. This courtroom image is so common and detrimental that I often explicitly deny its applicability.

Other metaphors help to structure therapy and clarify the couple's interpersonal process. I often invoke *dancing together* (with its nonverbal connotations of mutuality, power‐sharing, and potential for pleasure or embarrassment), *negotiation between countries* (when compromises or sacrifices must be made), or Freud's *archaeology* metaphor (describing the search for hidden or unconscious origins of problems).

When teaching students that they may need to raise avoided topics (such as a couple's absent sex life), I share a story about *my middle‐school dance instructor*. Seeing the boys and girls lined up on opposite sides of the room, he announced it was time to pick a partner and get started. This image highlights the therapist's role in facilitating necessary but uncomfortable conversations.

### 3.2 *Music and Words:* Distinguishing Between Process and Content

Once partners begin talking to each other, I focus on their maladaptive interpersonal processes: their *dances* or *vulnerability cycles* (Scheinkman and Fishbane 2004). All schools of couple therapy do this, though they differ in how they do so. Here, I point out the difference between the process and the content during couple disagreements by noting the difference between *the music* and *the words in songs*—where the music conveys emotion and can be distinguished from the words. Words still matter, of course, but body language and tone of voice often overshadow them, as these convey the speaker's emotional stance toward the listener.

While this distinction makes logical sense to most clients, many struggle with the idea that a “system” can have emergent properties—often destructive and amplifying ones—that cannot be blamed entirely on one person. The following metaphors help clients appreciate the systemic nature of their difficulties.

### 3.3 *Chemical Reactions:* Explaining Mutual Causation and Destructiveness

To illustrate how both partners typically contribute to relationship problems and to reduce mutual blaming, I compare partners to two harmless, colorless reagents in separate beakers. When mixed, these reagents can undergo a dramatic transformation, becoming explosively hot, ice cold, or foul‐smelling. One reagent might think, “I was just fine before—not hot, cold, or smelly. This sudden change, in which I don't recognize myself, must be the fault of that other damn chemical!” This metaphor vividly demonstrates that group process is not reducible to individual behaviour and is experience‐near for clients who are often feeling blamelessly victimized by their partner.

### 3.4 *Vicious Cycles and Their Punctuation:* Addressing “Who Started the Fight?”

When I describe *vicious cycles*—a universal metaphor in couple therapy already familiar to most clients—I note that people typically *punctuate* their narratives by beginning with a partner's perceived misdeed. Beth may say her anger at Fred stems from his failure to call the plumber, as he'd promised. Fred may grudgingly admit this but explain that he didn't call because he was angry that Beth hadn't had sex with him in 2 weeks. Beth may then reply that she hadn't been in the mood because Fred so frequently breaks promises about household tasks, like calling the plumber! I then affirm that both are right, but that their individual *punctuation*, their account of what started it all and when, is often arbitrary. We see these same circular arguments unfold in real time in our offices, where a cycle's starting point can feel experientially different to each partner.

### 3.5 *Hungry Diners and Unresponsive Waiters:* Normalizing Pursuit and Off‐Putting Demands

Escalation often involves one or both partners becoming increasingly loud, impatient, or aggressive—nagging, guilt‐tripping, or swearing. These ineffective attempts to influence a partner tend to occur and intensify when that partner appears unresponsive. Therapists can normalize such counterproductive behavior by framing it systemically. One metaphor I use involves a *hungry diner* trying to get the attention of an *unresponsive waiter*. At first, the diner waits politely. Then, he signals nonverbally. Next, he calls out calmly. Eventually, he yells. Often, both partners are better understood as *hungry diners* speaking to *unresponsive waiters*.

### 3.6 *Drowning Swimmers, Psychological Oxygen, and Attunement:* Panic and Anger When Needs Go Unmet

Like the *hungry diner*, the *drowning swimmer* metaphor illustrates the panic, inarticulateness, and possible counterintuitive combativeness (familiar to trained lifeguards) that can arise when people feel deprived of *psychological oxygen*. Most often, both partners resemble *drowning swimmers*, each desperate for rescue and attention, recalling Heinz Kohut's frequent use of *oxygen* as a metaphor for core relational needs (e.g., 1984).

The *drowning swimmer* metaphor is particularly useful when someone hasn't been heard—resembling the *squeaky wheel* that squeaks louder when *not getting the grease*; the *alarm clock* that *keeps repeating* when a sleeper fails to wake and turn it off; or when the *ignored driver* explodes in road rage. In all these cases, the missing ingredient is responsive *attunement*—a central metaphor of much contemporary psychotherapy—as when one *tunes an analog radio to the right station*. Such responsiveness—accurately acknowledging that a problem exists—most often helps restore calm.

### 3.7 *Micromanaging Bosses, Frightened Parents, and Coal Mine Canaries:* Unpleasant, Repetitive Control Due to Anxiety

While many pursuers *bang on the closed door* due to unmet needs for love, support, or companionship (Kohut's *psychological oxygen*), others, like some *micromanaging bosses*, do so out of anxiety (“If we don't make that deadline, we'll lose the deal!”). Clients who have worked in organizational settings get this metaphor, which distances them from the marital arena and normalizes the nagging of an anxious spouse (say, about their partner's need for medical attention). With other clients, I describe how *parents routinely yell at their children when they sense danger* (a child running into the street) or how my wife's acute sense of smell once detected a dangerous gas leak in a house we were visiting (a real *canary in a coal mine*).

### 3.8 *The Emperor and the Nightingale:* Damage From Micromanaging

While some micromanaging can be normalized, it can also be overdone and (ironically) lead to passive‐aggressive noncompliance. To make this point, I tell Hans Christian Andersen's story of the *Chinese Emperor* who learned he had to uncage his captured songbird, as she told him was necessary if she was to sing for him.

### 3.9 *Firefighters Battling Forest Fires, Castle Dwellers Pulling up Drawbridges:* Normalizing Distancing and Flight

Just as escalating angry pursuit can seem appropriate in some situations, so can flight. Withdrawal becomes more comprehensible and acceptable if one remembers that *firefighters facing a raging forest fire* must sometimes retreat temporarily. And *villagers under attack* from foreign enemies will understandably *retreat to their castles and pull up their drawbridges*. Helping clients share their reasons for retreating, rather than simplistically blaming retreat on “fears of intimacy,” frequently deepens the treatment. The *drawbridge* metaphor also points to circularity: If pursuers come in peace, distancers won't need to pull up their drawbridges.

### 3.10 *Sports Team‐Mates and Co‐Captains:* Some Challenges of Two‐Person Groups

Having played lots of doubles tennis, it's common for me to compare marriage to a *team sport*, where outcomes are shared and some degree of self‐sacrifice (“There's no I in TEAM!”) and forbearance (you must support your partner, even and especially after they've messed up) are required.

To maintain team cohesiveness, there is also the problem of agreeing on strategy and a course of action in a two‐person group (an almost universal issue in couple therapy), a situation I call *the co‐captains' problem*, and illustrate by noting how hard it would be if *a ship approaching an iceberg had two captains debating whether to turn to port or starboard*. For that reason, ships have only one captain, whereas equity in marriage requires that each partner *gets a vote* (another useful metaphor) and must accept the challenge of *two co‐captains*.

### 3.11 *Duets, Bands of Brothers, Collaborators*: Some Advantages of Two‐Person Groups

While being co‐captains can create problems, there are times when *two heads are better than one*, when performers *sing harmoniously in duets*, or when *soldiers feel a special bond of respect and protectiveness*. These metaphors can be used when stress increases—say, from work or children—requiring partners to collaborate and work together. Indeed, Dan Wile made *collaboration* the core metaphorical goal of his insightful couple therapy (Wile and Kaufmann 2021).

## 4 Psychodynamic Metaphors

Once problematic cycles have been identified, therapists can dig deeper into the psychology that powers them and causes relationship distress (Nielsen 2017b).

### 4.1 *The Yellowstone Park Cartoon*: Hidden Issues

**FIGURE 1:**
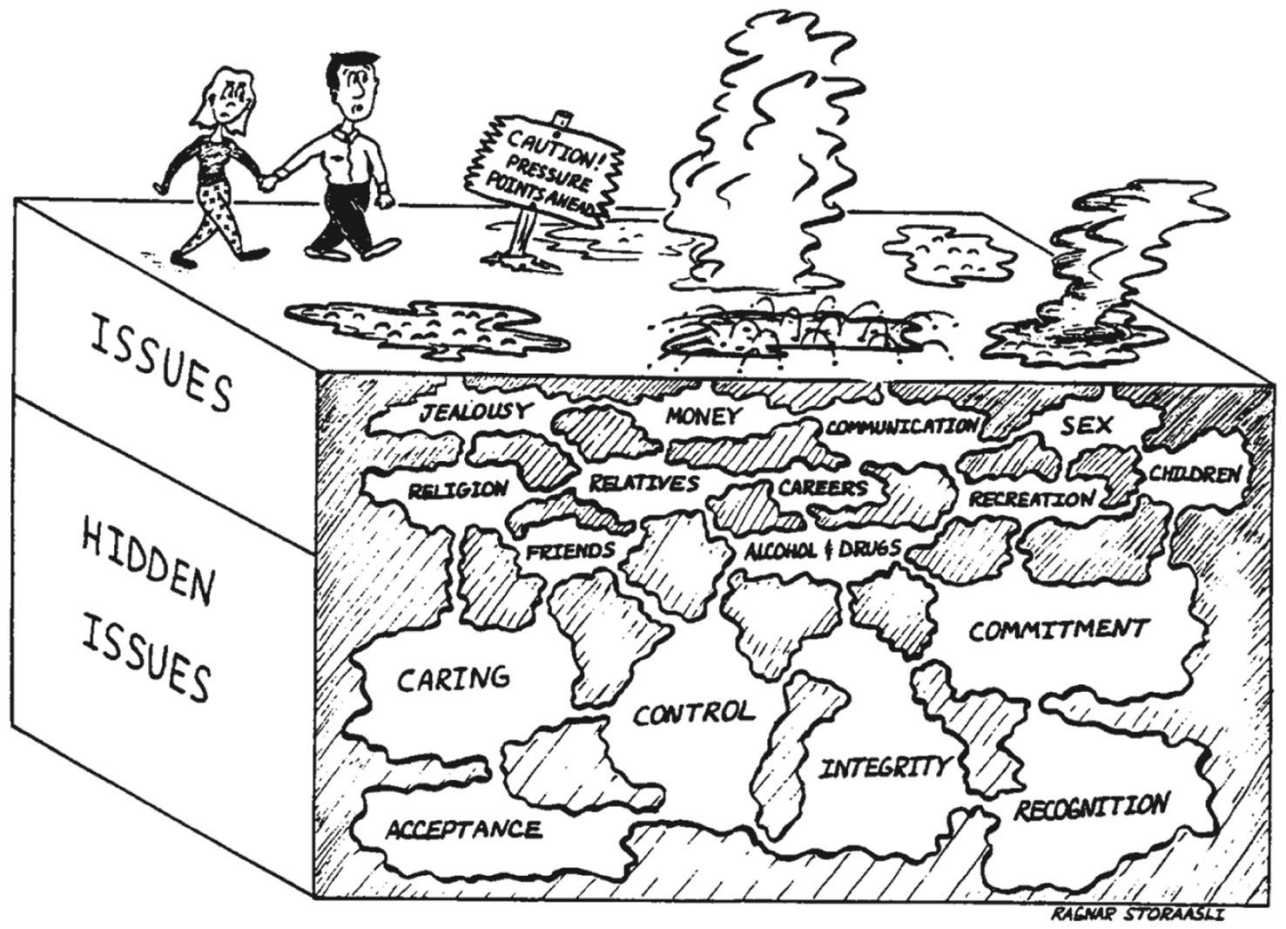
Yellowstone Park: Hidden (often unconscious) Issues. Used with permission from the book, *Fighting for Your Marriage* (2001) by Markman, Stanley, & Bloomberg. Artist: Ragnar Storaasli.

Figure 1 shows a couple proceeding through life, depicted as walking through a potentially dangerous Yellowstone Park. Like this couple, our couples encounter, often by surprise, a surface issue (a disordered dishwasher, an unexpected bill, or a visit from a difficult relative) that stirs something deeper, often unconscious and more meaningful (an issue concerning control, finances, commitment, or recognition). Early in therapy, I often share this picture to illustrate the central idea of *hidden issues*—namely, that significant couple disputes are never “about nothing” but usually involve very basic concerns, ones often outside conscious awareness.

### 4.2 *The House‐Moving Scene, TAT Cards, and the Rubin Vase*: Divergent Subjectivities

To illustrate how the same event can be experienced differently—a frequent source of couple conflict—I describe a scene from the film *The Story of Us*: A married couple on the verge of separation are driving their kids to summer camp when traffic is slowed by a house being moved. The parents each experience the house correctly, but differently. Mom sees it as an impediment, worries they will be late, and suggests finding a different route. Dad finds it an opportunity for levity at a time of family stress and tells a joke about someone pooping in the house's bathroom. The scene, an example of their polarization in other areas, ends with the couple staring silently, painfully, and disapprovingly at each other. When couples argue about “what really happened,” I use this movie scene to highlight the constructivist idea that many events do not come with fixed meanings.

**FIGURE 2:**
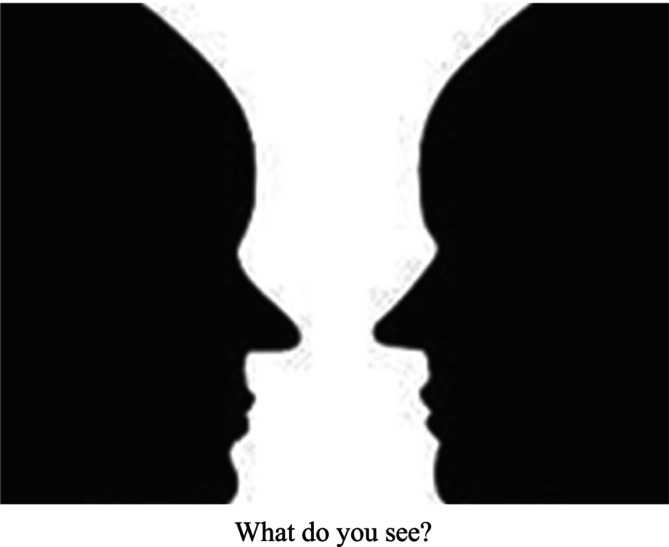
The Rubin Vase.

Other times, I make the same point by noting how people can react differently to the same film or Thematic Apperception Test (TAT) card. Sometimes I show couples the Rubin Vase image (Figure 2), ask what they see, and note that the correct answer is BOTH a vase AND two faces. These metaphors underlie my “You're both right” intervention, which validates the partners' divergent subjective realities. The TAT card metaphor also illustrates how clients jump to conclusions about their partners, either due to limited information or because they are triggered and agitated by repeated transference experiences (discussed in the next section).

### 4.3 *Allergies, Maps, Default Settings, Owners' Manuals, and Sore Shoulders:* Transference Sensitivities

To convey the concept of negative transference expectations that evoke intense, often idiosyncratic, emotional responses, I compare them to *childhood allergies* (e.g., to penicillin, peanuts, or bee stings). Just as someone who has had a prior bee sting may have a severe reaction to a subsequent sting, a person abandoned early in life may have an intense reaction to a spouse's business trip. I illustrate how such *allergies* follow from unconscious internal *maps* (if your map shows sea monsters, you'll avoid going sailing!) or the *default settings on a computer*. These *maps* and *settings* are often outside our awareness—in the computer metaphor because they were set at the factory; with clients because they originated in childhood. Until made conscious, they may seem inevitable as in “That's just the way life is.”

Clients' maps include not only images of the self and others interacting in traumatic scenarios but also beliefs about whether these can change and whether anyone will help, as they include expectations of resilience and attachment security. Therapy allows clients to test whether their maps still apply—whether history is repeating or will repeat—even when that feels risky, *as when Columbus literally sailed into uncharted waters*.

Couple therapy allows partners to learn each other's *allergies*, *maps*, and *settings*, valuable acquisitions, since, unlike cars, partners do not come with *owners' manuals*. And to foster empathy for these sensitivities, I remind clients that they would avoid slapping their *partner's sore shoulder* if they knew it had been injured.

### 4.4 *Turtles, Rabbits, Castles, and Home Remedies*: Defenses and Coping Mechanisms

Many metaphors normalize self‐protective defenses. In addition to the *retreating firefighters* mentioned previously, I often refer to *turtles* (hiding in their shells), *rabbits* (ever alert and ready to flee), and *castles* (also mentioned previously, with walls and drawbridges, which provoke partners to attack or withdraw). I use *home remedies* to encompass various coping styles—perfectionism, self‐blaming, heavy drinking, extramarital affairs—and mention their *side effects*, such as cirrhosis from alcoholism, partner loneliness from workaholism, and divorce from affairs.

### 4.5 *Oxygen, Basic Food Groups, and Love Languages*: Imaging Basic Interpersonal Needs

Helping marital partners meet each other's basic human needs for empathy, care, responsiveness, assistance, enthusiasm, and so on is central to a self psychological approach to couple therapy (Leone 2008). I have already cited the evocative metaphor of *oxygen* to characterize these needs. These can also be described as *basic food groups* (Shaddock 2000) or *love languages* (Chapman 1992), both of which help individualize what may be missing in particular relationships.

## 5 Behavioral/Psychoeducational Metaphors

While most couples do not expect to follow a specific script during “difficult conversations” (the term of art used by negotiation experts), teaching partners the value of structured plans and the importance of avoiding “what comes naturally” can significantly improve outcomes.

### 5.1 *Surgeons' Gloves, Pilots' Checklists, and Robert's Rules:* Following Rules Can Help

**FIGURE 3:**
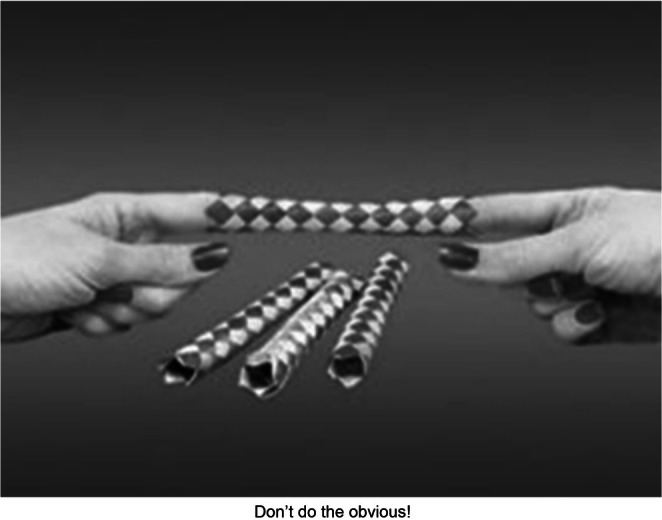
Chinese Finger Traps.

When couples object that following rules for difficult conversations feels unnatural, I sometimes agree and then remind them how *surgeons benefit from wearing gloves* and *pilots from using preflight checklists*. To show how instinctive reactions can backfire, I demonstrate a *Chinese Finger Trap* (Figure 3), where escape requires resisting the obvious response. But the metaphor I find most apt for couple work—one that normalizes the need for restraint to achieve better results—is *Robert's Rules of Order*, which I use to remind clients that successful business or committee meetings aren't free‐for‐alls.

### 5.2 *Wrestling Moves*: Value Naming Behavioral Options

Before I learned to wrestle in my high school gym class, my image of wrestling was simply two people grabbing each other and rolling around on the ground. But after I was taught time‐tested *named wrestling moves*, like a single‐leg takedown or a half Nelson, I quickly learned that these made an enormous difference. The same holds true for *couple wrestling*, where knowing specific conversational strategies such as using I‐statements or avoiding offering unsolicited advice can be immensely helpful.

### 5.3 *Appeals to Authorities, Dangerous Driving, Basketball Players, Public Speakers, Boxers, and Humor:* More Comparisons for Difficult Conversations

When I move on to teach best practices for speaking and listening, I appeal to authority, to commonsense similar situations, and to clients' sense of humor. I borrow the authority of John Gottman's research (Gottman et al. 1998) and The Harvard Negotiation Project (Fisher et al. 1981), often quoting Fisher's famous recommendation: “Be hard on the problem, soft on the person.”

I compare difficult conversations to *driving in dangerous conditions*—on icy roads, in the dark, or around sharp curves—and note how it helps to slow down and pay attention. For couples, this means “making a short story long” (Scheinkman and Fishbane 2004), as they explore (in therapy and later, on their own) hidden meanings, *hot buttons*, and unmet needs.

I remind clients that “failing to plan is planning to fail” and encourage them to know their ultimate goals (like a *basketball player aiming for the hoop* or a *golfer for the fairway*). I suggest that they can still function even when they are understandably anxious (like when *giving an important* speech or *serving for the match*) or when simultaneously anxious and angry (*like a boxer or soldier*).

Above all, I stress the importance of curiosity and empathic listening, sharing the humorous warning that *“The only people who listen when couples fight are… the neighbors!”*

### 5.4 *Personal Anecdotes, Myself as Satisfied Customer:* Providing Encouragement

Sometimes, when clients remain ambivalent about following my suggestions for better communication or problem solving, I share personal anecdotes to reinforce my message. I say, “When my wife Sheila and I go out, friends sometimes ask, ‘Sheila, we know Art teaches this stuff, but what's he really like during an argument?’ Most often, Sheila boosts my credibility by saying, ‘He turns into a different person; he changes gears.” I explain that I do this by following certain rules, especially becoming curious and prioritizing empathic listening. My point is that I am personally a *satisfied customer*, not just someone offering advice from the sidelines, spouting the latest self‐help advice.

### 5.5 *Gottman, Gardening, and Love Languages*: Encouraging Positive Interactions

Many couples I see have stopped giving each other compliments, hugs, or satisfying sex. In less extreme cases, they simply take each other for granted. As I work to revive positivity (Nielsen 2016, Chapter 14)—often by identifying each partner's *love language*—I sometimes cite Gottman's research finding that successful marriages are marked by a preponderance of positive interactions over negative ones. I also compare marriage to a *garden that needs ongoing care* or share a joke that illustrates the same principle: A (foolish) man told his wife, “I already told you once that I love you. If I change my mind, I'll let you know.”

## 6 Metaphorical Interventions: Intertwined Benefits

My interest in metaphors has developed organically over many years. Like others before me, I have found that metaphors not only make difficult concepts comprehensible, experience‐near, and memorable, but that, in various combinations, they can:

- Encourage hope and suggest alternatives. Anecdotes about others who have benefitted—including the therapist as a *satisfied customer*—and references to scientific research implying therapist expertise all point the way out of marital darkness. Metaphors and stories can inspire people to endure hardship, to try something new along the way to accomplishing challenging goals, or, by contrast, to accept and heal from painful experiences (Atwood and Levine 1991; Burns 2001).
- Normalize and convey empathy for off‐putting or shameful behavior. People who yell or don't want to talk have their reasons. Sharing our own struggles can reduce transferences to the therapist as judgmental. Marital jokes can universalize difficulties—as in the quip “The only people who listen are… the neighbors.” These approaches reduce shame and open the door to exploring underlying issues. In self psychological terms, metaphors often provide selfobject experiences of empathic *mirroring* and *twinship*, allowing clients to feel understood by and connected to others who have faced similar struggles.
- Increase the likelihood that interventions will be remembered. My clinical experience aligns with research by Martin et al. (1992) and resonates with Gottschall's (2012, 118) observation: “The world's priests and shamans knew what psychology would later confirm: if you want a message to burrow into the human mind, work it into a story.”
- Lighten the mood and *calm the waters* by displacing the action. As in play therapy or when referencing movies, most metaphors create psychological distance from the immediate moment, making room for therapeutic reflection (Combs and Freedman 1990; Haen 2020; Lankton and Lankton 1989; Torneke 2017). As Littman noted (Barker 1985, vii), “Direct teaching of behavioral laws and principles often meets with resistance… Soap operas, fables, and parables have the advantage that they tell us about ourselves in an indirect and thus more acceptable manner.”
- Heighten or reveal emotion or unconscious connections when clients are overly controlled, intellectualizing, or closed off. Rather than calming the waters, some metaphors—think drowning swimmers or retreating fire fighters—are often action‐packed. As many have suggested (e.g., Atwood and Levine 1991; Bucci 2001), such metaphors help generate emotional arousal, which is critical to therapeutic success in general (Lane et al. 2022), especially with trauma survivors (Stern 2009).

## 7 Technical Suggestions

The artful use of metaphors requires mixing and matching the elements just described—sometimes aiming to calm things down, other times to heat things up—while always granting our minds the freedom, which Ogden (1997) terms reverie, to search both our own minds and our clients' language to locate metaphors that not only empathically capture the moment but also facilitate therapeutic progress. In preparing this paper, I selected from a large storehouse of metaphors, choosing those I use most often and have proved therapeutic. While these are tried and true, others bubble up in the moment. These spontaneous metaphors—emerging from memories, movies, songs, history, or jokes—often surprise me, especially when they surface from my distant past. Yet when they do, they typically match the mood and issue at hand.

So, while I am offering specific suggestions—*tools for your therapeutic toolboxes*—you should feel free to use whatever metaphors may arise in the moment to illuminate specific issues as they occur. The most effective metaphors align with your clients' language and life experiences—recalling their professions (machinist, lawyer, doctor, teacher, musician), hobbies (dancer, hiker, golfer, gardener), or experiences (world traveler, political advocate, school board member). Ideal metaphors fit the person's history, culture, and religious beliefs. When working cross‐culturally or across generation gaps, the best metaphors often come from the other person's culture or generation, even as some metaphors, like marriage as a shared journey, seem universal. In addition to that journey metaphor, a Japanese study (Zhou et al. 2017) found marriage represented by a physical union, a joint creation, a business, a pair of shoes, and tea (because tea can be simultaneously bitter and pleasant) and a Chinese study (Dunn 2004) added the image of chopsticks (suggesting equal and separate partners working collaboratively toward a common goal).

To help interventions stick, I often show couples the images I've referenced in this paper. If they resonate, I hand or email them copies. Some couples tape them up on their refrigerators.

Of course, I do not share every image that comes to mind, and I sometimes alter the cast of characters in personal stories or draw from current events, literature, or film. While such examples may feel safer, they are often less powerful in mitigating shame and fostering connection. Similarly, I may think of a joke that reflects some combination of my countertransference and the couple's psychology but choose not to share it. While not suited for sharing, such free associations frequently give me insight into what's happening in the moment or what may need attention later. And some clients don't want to hear stories—personal or otherwise—so I tailor my words to match their receptivity.

Many clients generate their own telling metaphors. For example, a couple I described in detail in Nielsen (2024) gave their repetitive fights a humorous, Monty Python‐inspired name: Reginald—thereby fulfilling Dan Siegel's metaphorical mantra, “Name it to tame it!” (Siegel and Bryson 2011). This humorous naming allowed them to slow down, change gears, and use the skills and understanding they had gained from therapy. Another client spoke of a relative who had recently stopped wearing a hairpiece to conceal his baldness—an act my client admired and later referenced in our work to help him become more self‐confident and emotionally open with his wife.

While research suggests that it does not much matter who originates a metaphor in therapy, co‐created ones often become useful themes that run through successful treatments (McMullen and Tay 2023; Torneke 2017). One such example from my practice began when it occurred to me to compare a man's inability to let go of a self‐destructive relationship to an eagle grasping a fish that was too heavy to carry—resulting in the eagle drowning. I knew the client was an avid fisherman, and he responded that he had indeed seen such a thing. A deeply committed Jew, he then deepened the metaphor, connecting it to a much darker image: Jews in Nazi Germany who failed to recognize the danger in time and then lost their lives. Like the metaphors of Reginald and the hairpiece, this powerful metaphor became part of our shared language.

Some painful or tragic metaphors originated by clients, like some of their nightmare dream images, become transformed in therapy. This process is most evident in Papp's examples (Papp 1983; Papp et al. 2013) of couple sculpting, where, for instance, a King Kong/Fay Wray couple and a policeman/criminal couple became less polarized and happier. But such transformations also arise spontaneously. One client of mine who described herself as “the family dog”—loved but not prioritized—gradually gained full status and came to see herself, and to be seen, as a co‐equal adult partner.

## 8 Discussion

As discussed by Summers (2013):

The linguists Lakoff and Johnson (1980) have argued convincingly that metaphor is not just a figure of speech used primarily by poets and fiction writers, but a way of thinking built into our conceptual system… To use Lakoff and Johnson's most discussed illustration: “Argument is war” shows that we think of argument the way we think of war: trying to gain ground, defending our position, seeing the other's position as indefensible. (p. 69)

The metaphor *argument is war* could sadly be applied to many couples who seek therapy. My metaphor of couple therapy as resembling *lessons* is much more hopeful. More broadly, following Lakoff and Johnson, we can see that metaphors are not merely figures of speech; they *saturate* our language and thought, and some are particularly helpful for illuminating therapeutic dynamics that are otherwise difficult to grasp. The metaphors presented in this paper bring life to some concepts that most of us find challenging, including: (a) that much of human experience is co‐created, nonlinear, and emergent rather than simply driven by one partner acting on the other; (b) that the meaning of events is often hidden, subjective, divergent, and rooted in early life sensitization; and (c) that many people are unaware of the need to employ communication rules with intimate partners and are unfamiliar with practical methods for improving their interactions.

Of course, these insights are no news to readers of this journal. Yet because partners routinely misattribute their difficulties to a recalcitrant or malevolent “other,” and can *fight to the death* over what “really happened,” the metaphors discussed here are especially useful in helping couple therapists address co‐created, circular causation. By illustrating that “it takes two to tango,” these images—blameless chemical reagents, hungry diners with inattentive waiters, retreating firefighters—help identify a couple's interactional process as the core issue, in what Michael White (2007) famously called an “externalizing conversation.”

Psychodynamic insights become more vivid and memorable when therapists describe how individuals interpret the same movie, the Rubin Vase, or a TAT card differently, and when they liken transference sensitivities to childhood allergies or default computer settings.

Finally, the need for structure in difficult conversations can be *jump‐started* with metaphors drawn from other domains: surgeons wearing gloves, pilots using preflight checklists, and most business meetings following Robert's Rules of Order.

## 9 Conclusion

Since ancient times, parables have helped make relationship challenges universal and memorable for us story‐telling animals. Like the parable of the Good Samaritan, many urge us to love our neighbors even when doing so is difficult. I hope this paper has provided you with a set of ready‐made metaphors and inspired you to create your own. With help from your clients, I encourage you to co‐create metaphors that are jargon‐free, energizing, and empathic—images that illuminate what blocks couples' progress and what might support their journey toward becoming more self‐aware and mutually supportive partners.

## Conflicts of Interest

The author declares no conflicts of interest.

## Data Availability

The author has nothing to report.

## Endnotes

^1^
For a wonderful paper illustrating the value of such a prescriptive metaphor to help patients overcome their fear of vaccinations, see Trogen (2017). The metaphor used—vaccination with a viral component, *not* the dangerous complete virus, results in a “viral fingerprint” that later will allow the body to identify and combat a real intruding viral criminal—fits both categories as it targets a patient problem and offers help by explaining a mechanism of cure.

^2^
I have italicized each metaphor under discussion, as here, with *Lessons*. Additionally, to show how pervasive metaphors are in everyday discourse, I have italicized others that appear along the way in this paper.
